# Carcinoembryonic antigen as a predictor of treatment outcomes in cancer patients receiving immune checkpoint inhibitors

**DOI:** 10.1080/07853890.2025.2531255

**Published:** 2025-07-25

**Authors:** Wangbin Ma, Qiao Shi, Xiaozhe Su, Lilong Zhang, Chen Chen, Chao Zhang, Ying Wang

**Affiliations:** ^a^ Department of General Surgery, Renmin Hospital of Wuhan University, Wuhan, China; ^b^ Laboratory of General Surgery, Renmin Hospital of Wuhan University, Wuhan, China; ^c^ Hubei Key Laboratory of Digestive System Disease, Wuhan, China; ^d^ Department of Urology, Renmin Hospital of Wuhan University, Wuhan, China; ^e^ Department of Traditional Chinese Medicine, Renmin Hospital of Wuhan University, Wuhan, China; ^f^ Department of Gastroenterology, Renmin Hospital of Wuhan University, Wuhan, China

**Keywords:** Carcinoembryonic antigen, immune checkpoint inhibitors, cancer, prognosis, immune response

## Abstract

**Objective:**

Carcinoembryonic antigen (CEA) is a widely used tumor marker and is associated with traditional therapeutic efficacy. Our study aims to assess the predictive significance of baseline carcinoembryonic antigen (CEA) levels and CEA level changes on the efficacy of immune checkpoint inhibitors (ICIs) in cancer patients.

**Methods:**

A systemic literature search was conducted in three digital repositories—Embase, PubMed, and the Cochrane Library—to obtain studies linking CEA with clinical results in cancer patients receiving ICIs from the year of inception of each database until 20 August 2024. Studies were included if they involved cancer patients treated with ICIs, assessed the prognostic significance of baseline CEA levels or CEA level changes, and reported at least one outcome metric, including overall survival (OS), progression-free survival (PFS), disease control rate (DCR), pathological complete response (pCR), or objective response rate (ORR). Duplicate studies were identified and removed using Covidence software following Cochrane collaboration guidelines. The Newcastle-Ottawa Scale was applied to evaluate study quality. Pooled hazard ratios (HRs) for OS and PFS, as well as odds ratios (ORs) for DCR, pCR, and ORR, were calculated with 95% confidence intervals (CIs).

**Results:**

The analysis included 27 studies, comprising a total cohort of 3662 patients. The findings revealed that cancer patients receiving ICIs with lower CEA levels had significantly improved OS (HR: 1.84, *p* < 0.001) and PFS (HR: 1.64, *p* < 0.001), along with higher DCR (OR: 1.81, *p* = 0.001), ORR (OR: 0.53, *p* = 0.001), and pCR (OR: 0.58, *p* < 0.001) compared to those with elevated CEA levels. Additionally, a reduction in CEA levels during immunotherapy was significantly associated with prolonged OS (HR: 0.507, *p* < 0.001) and PFS (HR: 0.501, *p* < 0.001), as well as increased ORR (OR: 2.39, *p* = 0.005) and DCR (OR: 2.94, *p* < 0.001).

**Conclusion:**

The results advocate for integrating CEA level assessments into the prognostic analysis for cancer patients.

## 1. Introduction

Immune checkpoints are essential components of the immune system, playing a key role in preventing inflammation caused by excessive T-cell stimulation and other roles [1]. Conversely, immunotherapy utilizes the body’s innate immune responses to both inhibit and target the proliferation of cancer cells. Immune checkpoint inhibitors function by interrupting these immune checkpoints, reinstating the capacity of T cells to identify and target cancer cells, thus surmounting the suppression of the immune system in the tumor’s microenvironment [1]. The method is demonstrated through employing monoclonal antibodies aimed at cytotoxic T lymphocyte-associated antigen (CTLA-4), programmed cell death ligand 1 (PD-L1), and programmed cell death protein 1 (PD-1) [2].

Immune checkpoint inhibitors (ICIs), whether administered as monotherapy or in conjunction with adjunctive treatments, such as chemotherapy and anti-angiogenic agents, have emerged as pivotal modalities in the management of various malignancies. Despite their transformative potential, the therapeutic benefit of ICI monotherapy remains limited, with response rates averaging ∼23% among patients with advanced solid tumors [3]. Moreover, a subset of patients may experience adverse effects, such as hyper-progression or severe toxicity [4]. Precision and reliability in biomarker selection are critical for identifying patients likely to respond positively to immunotherapy. Currently, the Food and Drug Administration (FDA) most commonly recognizes PD-L1 expression and tumor mutation burden (TMB) as key biomarkers in forecasting the success of immunotherapy [5]. Nonetheless, some patients with negative PD-L1 expression or low TMB levels still demonstrate therapeutic responses to immunotherapy. Additional potential predictive biomarkers, such as the microbiome, tumor-infiltrating lymphocytes, genetic signatures, and multi-omics profiling [6], face challenges related to high costs, procedural complexity, and dependence on tissue samples, which limit their clinical utility. Therefore, there is an urgent need to develop more cost-effective and practical biomarkers to better identify patient populations who stand to benefit most from immunotherapeutic treatments.

Carcinoembryonic antigens (CEAs) are glycosylphosphatidylinositol-anchored glycoproteins predominantly expressed in carcinomas of the gastric, pancreatic, lung, breast, and medullary thyroid tissues. CEA is widely recognized as a crucial biomarker in the diagnosis of CRC [7]. Recent research has demonstrated their utility as predictors of the efficacy of targeted therapies [8,9], chemotherapy [10,11], and surgical interventions [10,11]. However, their predictive value in the context of ICI therapy remains uncertain.

Hence, this research aims to comprehensively integrate current data and assess the predictive value of CEA levels in cancer patients receiving immunotherapy.

## 2. Methods

This examination adhered to the Preferred Reporting Items for Systematic reviews and Meta-Analyses (PRISMA) standards (Table S1) and was recorded in the PROSPERO International Prospective Register of Systematic Reviews [12].

### 2.1. Search strategy

As of 20 August 2024, a digital exploration was carried out in various bibliographic repositories, such as PubMed, EMBASE, and the Cochrane Library. The investigation employed distinct keywords including: ‘immune checkpoint inhibitors’ [Mesh], ‘ICIs’, ‘PD-1 Inhibitors’, ‘PD-L1 Inhibitors’, ‘CTLA-4 Inhibitors’, ‘Pembrolizumab’, ‘Ipilimumab’, ‘Atezolizumab’, ‘Nivolumab’, ‘Camrelizumab’, ‘Sintilimab’, ‘Tislelizumab’, ‘Toripalimab’, ‘Envafolimab’, ‘Carcinoembryonic Antigen’ [Mesh], and ‘CEA’. Furthermore, unpublished research data search was conducted using Google Scholar, followed by a manual review of the reference lists for qualified studies. Following Cochrane’s collaborative guidelines, Covidence software was employed to amalgamate results from manual and digital sources, thereby improving the efficiency of data management.

### 2.2. Inclusion and exclusion criteria

Specific inclusion criteria were established to guide article selection: (i) studies involving patients diagnosed with cancer; (ii) use of ICIs as the therapeutic intervention; (iii) assessment of the prognostic significance of baseline CEA levels (before ICI administration) and CEA level changes (during ICI treatment); and (iv) documentation of at least one of these outcome metrics: overall survival (OS), progression-free survival (PFS), disease control rate (DCR), pathological complete response (pCR), objective response rate (ORR) [13].

Exclusion criteria included: (i) studies based on animal research, literature reviews, case studies, or conference abstracts; (ii) studies lacking hazard ratios (HRs) or odds ratios (ORs) for outcome assessment from text or published data; and (iii) studies where baseline CEA data were treated as continuous variables. In cases of overlapping patient cohorts, preference was given to studies providing comprehensive data and employing robust methodologies [13].

### 2.3. Data extraction and quality assessment

Throughout the data gathering phase, essential information was gathered, encompassing the author, year of publication, design of the study, duration of the study, geographical area of the study, type of cancer, treatment methods, number of samples, age, and the results (whether cut-off or control). The primary sources for Hazard ratios (HRs), odds ratios (ORs), and their respective 95% confidence intervals (CIs) were multivariate analyses; in their absence, they came from univariate analyses or were derived from survival charts using Engauge Digitizer software. Observational study quality was evaluated through the Newcastle-Ottawa Scale (NOS), considering studies with a score of six or above as of high quality [14]. A total of nine quality standards were distributed among areas including choosing participants, ensuring comparability, and evaluating outcomes. Each step, including retrieving literature, screening, extracting data, and assessing quality, was independently executed by a trio of researchers, resolving any inconsistencies by consulting the lead author.

### 2.4. Statistical methods

The statistical evaluation was performed with Stata 15.0, and the results were depicted using forest plots. To assess heterogeneity, Cochran’s *Q* test and *I*^2^ statistics were employed, defining significant heterogeneity as a *p*-value below 0.1 and an *I*^2^ value exceeding 50% [13]. When faced with considerable diversity, the DerSimonian-Laird method was employed for a random-effects model; in other scenarios, the Inverse Variance method was utilized for a fixed-effects model. The evaluation of publication bias was conducted through the application of Egger’s regression test and Begg’s test [13,15]. To confirm the solidity of the findings, sensitivity analyses were conducted by methodically omitting each study. Analyses of subgroups were carried out utilizing cancer type, cut-off values, and Cox model. The threshold for statistical relevance was set at a two-tailed *p*-value < 0.05.

## 3. Results

### 3.1. Search results and included studies

**Figure 1.**
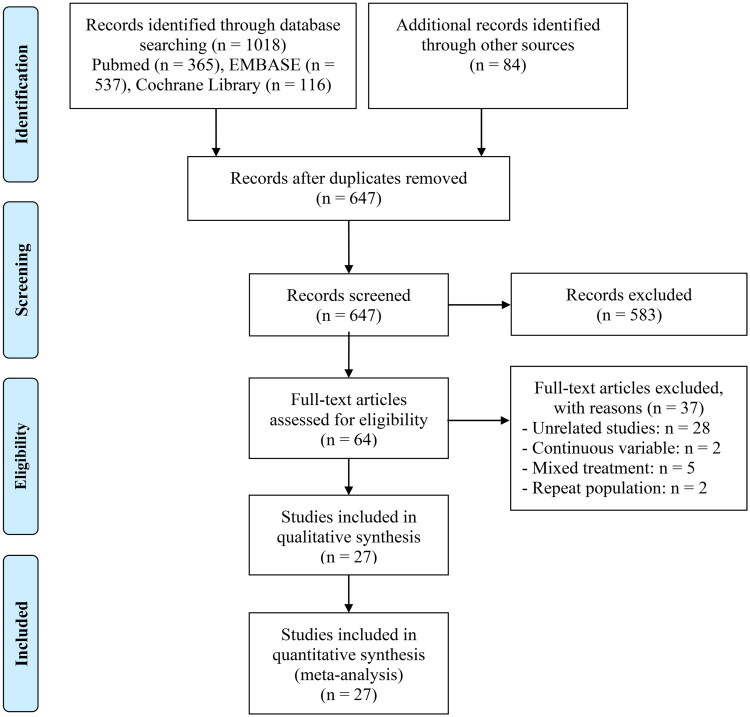
The flow diagram of identifying eligible studies.

Utilizing a predefined search approach and a hands-on examination, 1102 articles were pinpointed as possibly pertinent. Out of the total, 455 duplicates were eliminated, and 583 were discarded due to their failure to satisfy the selection standards related to their titles and abstracts. Following an extensive examination of the complete texts in the last 64 articles, 37 were omitted due to their non-compliance with the predefined criteria. As a result, 27 studies [16–42] were considered suitable for inclusion (Figure 1).

### 3.2. Study characteristics

Table 1 displays the principal features of the research incorporated in this study. The research included 4433 patients in total, with males constituting 72.65% of them. Average or median ages spanned between 58 and 70 years, with sample sizes ranging from 20 to 716 individuals. The majority of the research (21 out of 27) took place in China, followed by two in Japan, two in Italy, a single one in Qatar, and a Multinational. Each study was conducted retrospectively, obtaining scores between 6 and 8 on the Newcastle-Ottawa Scale (NOS), suggesting a minimal risk of bias (Table 1).

**Table 1.** Main characteristics of the studies included.

Study	Study design	Study period	Study region	Cancer type	Treatments	Sample size	Age	Gender (male/female)	Outcome (cut-off or control)	NOS
An et al. 2024	R	10/2015–10/2022	China	Biliary tract cancers	Sintilimab, camrelizumab, tislelizumab	39	59 (45–77)^b^	22/17	OS, PFS (5 ng/ml)	7
Zhu et al. 2023	R	06/2020–09/2022	China	ICC	Toripalimab	53	58 (51–66)^a^	33/20	OS, PFS (5 ng/ml)	7
Yang et al. 2022	R	05/2019–08/2021	China	ICC	Camrelizumab, sintilimab	20	59 (34–76)^b^	11/9	OS, PFS (5 ng/ml)	6
Yang et al. 2023	R	03/2017–04/2022	China	Biliary tract cancers	Camrelizumab, sintilimab, nivolumab, pembrolizumab	31	61.0 ± 11.8^c^	19/12	OS, PFS, ORR, DCR (7.2 ng/ml)	7
Cheng et al. 2024	R	01/2016–12/2020	China	Gallbladder cancer	ICIs	69	39^d^	33/36	OS, PFS, DCR (100 U/ml)	8
Raza et al. 2023	P	09/2020–07/2022	Qatar	NSCLC	Pembrolizumab, nivolumab, durvalumab	31	59 (40–80)^b^	26/5	PFS (1.6 ng/ml)	7
Huang et al. 2020	R	08/2016–12/2018	China	NSCLC	Nivolumab, pembrolizumab, atezolizumab, ipilimumab	61	11^e^	38/23	OS, PFS, ORR (5 ng/ml)	7
Liu et al. 2023	R	01/2019–12/2021	China	HCC	ICIs	151	57.41 ± 9.14^c^	124/21	OS, PFS (2.4 ng/ml)	8
Sun et al. 2022	R	01/2018–01/2020	China	NSCLC	Nivolumab, camrelizumab, tislelizumab	79	61 (40–77)^b^	63/16	pCR (5 ng/ml)	7
Kataoka et al. 2018	R	01/2016–11/2016	Japan	NSCLC	Nivolumab	189	49^f^	139/50	OS, PFS (13.8 ng/ml)	7
Wang et al. 2023	R	01/2019–07/2021	China	Gastric cancer	Nivolumab, pembrolizumab, or toripalimab.	249	–	145/105	ORR (5ng/ml)	8
Dall’Olio et al. 2020	R	08/2015–01/2020	Italy	NSCLC	Nivolumab, atezolizumab, pembrolizumab	207	69.54^g^	142/65	OS, DCR (8 ng/ml); OS, DCR (CEA reduction ≥ 20%)	7
Wen et al. 2022	R	08/2018–01/2021	China	NSCLC	PD-1 inhibitor	90	–	69/21	OS, PFS, ORR, DCR (CEA increase)	7
Shen et al. 2022	R	07/2016–01/2020	China	NSCLC	Pembrolizumab	266	173^d^	218/48	OS, PFS (5 ng/ml)	8
Huang et al. 2023	R	07/2017–07/2021	China	NSCLC	Pembrolizumab, nivolumab, atezolizumab, sintilimab, camrelizumab, tislelizumab	716	61.10 ± 10.55^c^	611/105	OS, PFS, ORR, DCR (5 ng/ml)	8
Zhang et al. 2023	R	09/2020–04/2022	China	Cholangiocarcinoma	Sintilimab, camrelizumab, pembrolizumab, toripalimab, tislelizumab	57	–	30/27	OS, PFS (5 ng/ml)	8
Chen et al. 2021	R	08/2018–12/2019	China	NSCLC	Pembrolizumab, sintilimab, toripalimab	151	63 (54–69)^a^	115/36	ORR, DCR (3.5 ng/ml); OS, PFS, ORR, DCR (CEA increase)	7
Chen et al. 2023	R	02/2019–06/2021	China	Pancreatic cancer	Pembrolizumab, nivolumab, toripalimab, sintilimab, tislelizumab, camrelizumab	27	64 (46–77)^b^	18/9	OS, PFS (5 ng/ml)	7
Shirasu et al. 2018	R	12/2015–09/2016	Japan	Lung adenocarcinoma	Nivolumab	50	64.5 (39–76)^b^	31/19	PFS (5 ng/ml)	7
Liu et al. 2023	R	05/2019–07/2022	China	NSCLC	ICIs	116	59.7 ± 7.8^c^	108/8	pCR (5 ng/ml)	7
Du et al. 2023	R	08/2018–05/2021	China	NSCLC	Pembrolizumab, sintilimab, toripalimab, camrelizumab, tislelizumab	146	74^e^	111/35	OS, PFS, DCR (29.9 ng/ml); OS, PFS (CEA increase)	8
Dal Bello et al. 2019	R	05/2015–05/2016	Italy	NSCLC	Nivolumab	70	70 (44–85)^b^	48/22	OS, PFS (5 ng/ml); OS, PFS, DCR (non-CEA reduction ≥ 20%)	7
Gao et al. 2023	P	–	China	Rectal cancer	Tislelizumab	26	60.5 ± 11.8^c^	14/12	pCR (5 ng/ml)	7
Yang et al. 2023	R	07/2017–07/2021	China	NSCLC	Pembrolizumab	716	61.10 ± 10.55^c^	611/105	OS, PFS, DCR (non-CEA reduction ≥ 20%)	8
Duan et al. 2024	R	01/2018–12/2022	China	Gastric cancer	Nivolumab, pembrolizumab, cedilimumab, carrelizumab, tislelizumab, toripalimab	105	55 (27–85)^b^	51/54	OS, PFS	7
Li et al. 2023	P	01/2019–12/2020	China	Gastric cancer	Camrelizumab	52	57^h^	35/17	pCR (4.7 ng/ml)	6
Rimini et al. 2024	R	02/2022–01/2024	Multinational	Biliary tract cancer	Durvalumab	666	67 (29–89)^b^	355/311	OS, PFS	8

R: retrospective study; P: prospective study; ICI: immune checkpoint inhibitors; NSCLC: non-small cell lung cancer; HCC: hepatocellular carcinoma; ICC: intrahepatic cholangiocarcinoma; PD-1: programmed cell death receptor protein 1; OS: overall survival; PFS: progression-free survival; ORR: objective response rate; DCR: disease control rate; CEA: carcinoembryonic antigen.

^a^Median with interquartile range.

^b^Median with range.

^c^Mean ± standard deviation.

^d^Age ≥60 years.

^e^Age ≥65 years.

^f^Age ≥75 years.

^g^Median age.

### 3.3. Baseline CEA levels and OS

**Figure 2.**
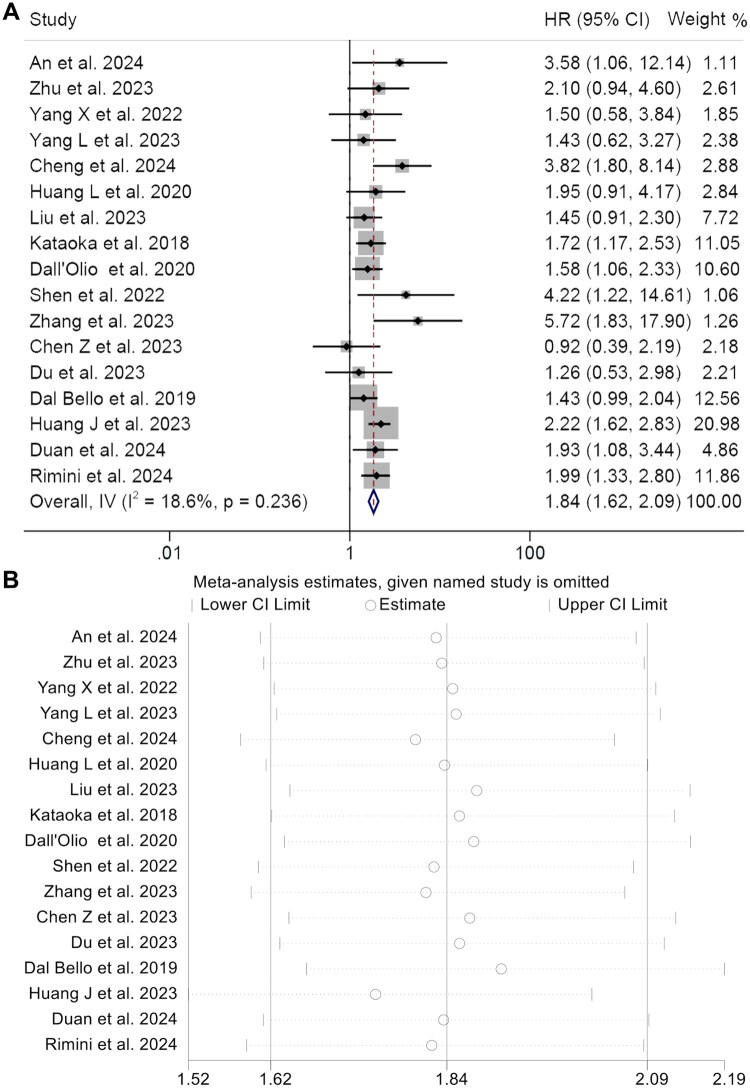
Forest plots of the relationship between CEA and overall survival in all included studies (A). Sensitivity analysis of the association between CEA and overall survival (B). HR: hazard ratio; CL: confidence interval. Meta-analysis pooling of aggregate data using the common-effect inverse-variance model.

In our study, we incorporated 17 research projects with 2873 patients to assess how CEA levels affect overall survival in cancer patients undergoing ICIs. Findings indicated a notable correlation between higher CEA levels and a markedly reduced overall survival (HR: 1.84, 95% CI: 1.62–2.09, *p* < 0.001, Figure 2A) in contrast to lower CEA levels. Evaluating heterogeneity through Cochran’s Q test and I^2^ statistics revealed notable heterogeneity (*I*^2^ = 18.6%, *p* = 0.236), prompting the adoption of a fixed-effects model. The stability and solidity of the combined HRs for OS were verified through sensitivity analyses that systematically omitted each study (Figure 2B). Analysis of publication bias using Begg’s and Egger’s tests showed no notable publication bias (Egger’s test: *p* = 0.592, Begg’s test: *p* = 0.431) and Funnel plot shows that the studies on both sides are basically the same, so there is no obvious publication deviation (Figure S1A).

To delve deeper into the predictive significance of CEA, analyses of subgroups were performed, focusing on cancer types, threshold values, and the Cox model. The studies consistently found a connection between higher CEA levels and reduced OS in both multivariate (HR = 1.87, 95% CI: 1.57–2.21, *p* < 0.001) and univariate studies (HR = 1.81, 95% CI: 1.49–2.20, *p* < 0.001). Higher CEA was correlated with poorer overall survival in various cancers, notably gastrointestinal cancer (GTC) (HR = 1.91, 95% CI: 1.55–2.35, *p* < 0.001) and non-small cell lung cancer (NSCLC) (HR = 1.80, 95% CI: 1.53–2.12, *p* < 0.001). Fluctuations in the threshold values for CEA had no impact on the relationship between CEA and OS in patients treated with ICIs. Moreover, the same results hold true in different countries (Table 2).

**Table 2.** Subgroup analysis of the association between carcinoembryonic antigen and the outcomes of cancer patients treated with immune checkpoint inhibitors.

Variable	Included studies	Test of association			Test of heterogeneity		
		HR	95%CI	*p*-Value	Modal	*I* ^2^	*p*-Value
Cox model (OS)							
Univariate analysis	8	1.81	1.49–2.20	***p* < 0.001**	F	0	*p* = 0.470
Multivariate analysis	9	1.87	1.57–2.21	***p* < 0.001**	F	38.4%	*p* = 0.112
Cut-off (OS)							
5 ng/ml	11	1.95	1.59–2.39	***p* < 0.001**	R	21.5%	*p* = 0.238
>5 ng/ml	4	1.59	1.24–2.05	***p* < 0.001**	R	0	*p* = 0.918
Other	2	2.24	0.87–5.79	*p* = 0.095	R	78.4%	*p* = 0.031
Cancer type (OS)							
GTC	10	1.91	1.55–2.35	***p* < 0.001**	F	29.5%	*p* = 0.174
NSCLC	7	1.80	1.53–2.12	***p* < 0.001**	F	10.6%	*p* = 0.349
Country (OS)							
China	13	1.99	1.59–2.49	***p* < 0.001**	F	24.9%	*p* = 0.192
Italy	2	1.50	1.15–1.95	***p* = 0.003**	F	0	*p* = 0.713
Other	2	1.86	1.42–2.42	***p* < 0.001**	F	0	*p* = 0.594
Cox model (PFS)							
Univariate analysis	11	1.49	1.19–1.87	***p* = 0.001**	R	48.8%	*p* = 0.034
Multivariate analysis	7	2.15	1.41–3.26	***p* < 0.001**	R	66.0%	*p* = 0.007
Cut-off (PFS)							
5 ng/ml	12	1.59	1.28–1.97	***p* < 0.001**	R	57.2%	*p* = 0.007
>5 ng/ml	3	1.41	1.02–1.95	***p* = 0.039**	R	0	*p* = 0.397
<5 ng/ml	3	3.16	1.04–9.59	***p* = 0.042**	R	81.1%	*p* = 0.005
Cancer type (PFS)							
GTC	10	1.52	1.21–1.91	***p* < 0.001**	R	43.4%	*p* = 0.069
NSCLC	7	1.94	1.53–2.46	***p* < 0.001**	R	27.0%	*p* = 0.223
Country (PFS)							
China	13	1.71	1.34–2.17	***p* < 0.001**	R	49.4%	*p* = 0.022
Other	5	1.48	1.14–1.91	***p* = 0.003**	R	42.2%	*p* = 0.140

HR: hazard ratio; CL: confidence interval; OS: overall survival; PFS: progression-free survival; GTC: gastrointestinal cancer; NSCLC: non-small cell lung cancer; R: random-effect model; F: fixed-effect model. Bold values are *p* < 0.05.

### 3.4. Baseline CEA levels and PFS

**Figure 3.**
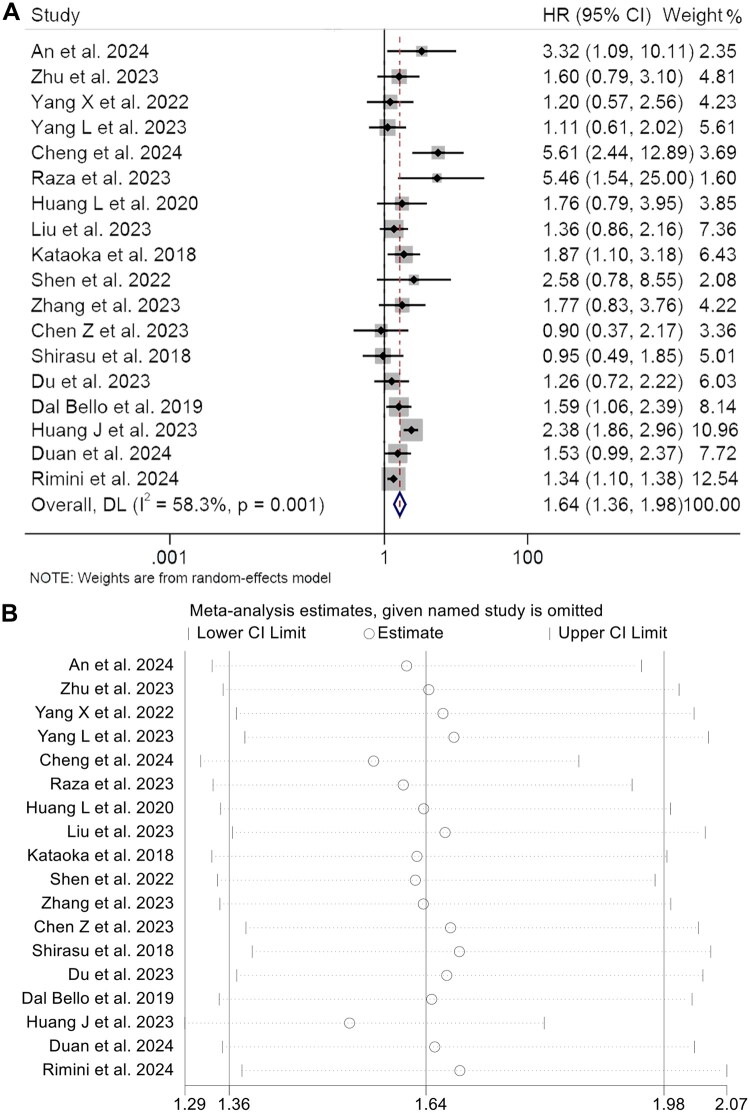
Forest plots of the relationship between CEA and progression-free survival in all included studies (A). Sensitivity analysis of the association between CEA and progression-free survival (B). HR: hazard ratio; CL: confidence interval. Meta-analysis pooling of aggregate data using the random-effects inverse-variance model with DerSimonian-Laird estimate of tau [2].

This research involved examining 18 studies with 2747 patients to evaluate how varying CEA levels affect PFS in cancer patients undergoing ICIs treatment. The findings showed that individuals with lower CEA levels had a notably extended PFS duration (HR: 1.64, 95% CI: 1.36–1.98, *p* < 0.001, Figure 3A) in contrast to those with higher CEA levels. Evaluating heterogeneity through Cochran’s *Q* test and *I*^2^ statistics showed significant heterogeneity (*I*^2^ = 58.3%, *p* = 0.001), leading to the adoption of a random-effects model. The stability and strength of the combined hazard ratios for PFS were verified through sensitivity analyses that systematically omitted each study (Figure 3B). Analysis of publication bias through Begg’s and Egger’s tests revealed an absence of notable bias (Egger’s test: *p* = 0.214, Begg’s test: *p* = 0.198) and Funnel plot shows that the studies on both sides are basically the same, so there is no obvious publication deviation (Figure S1B).

Analyses of subgroups reveal a steady link between higher CEA levels and reduced PFS, evident in both multivariate (HR = 2.15, 95% CI: 1.41–3.26, *p* < 0.001) and univariate studies (HR = 1.49, 95% CI: 1.19–1.87, *p* = 0.001). Additionally, higher CEA levels correlated with notably poorer PFS in various cancer types (GTC: HR = 1.52, 95% CI: 1.21–1.91, *p* < 0.001; NSCLC: HR = 1.94, 95% CI: 1.53–2.46, *p* < 0.001). Fluctuations in CEA threshold values had no effect on the relationship between CEA and PFS among patients treated with ICIs. Moreover, the same results hold true in different countries (Table 2).

### 3.5. Baseline CEA levels and ORR and DCR and pCR

**Figure 4.**
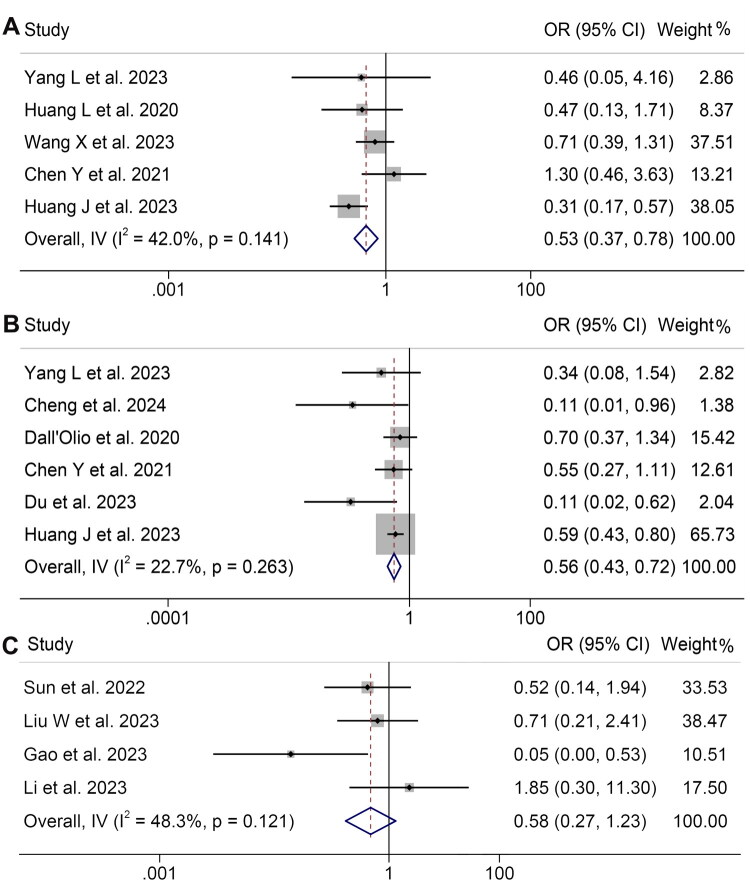
Forest plots of the relationship between CEA and objective response rate in all included studies (A). Forest plots of the relationship between CEA and disease control rate in all included studies (B). Forest plots of the relationship between CEA and pathological complete response in all included studies (C). HR: hazard ratio; CL: confidence interval. Meta-analysis pooling of aggregate data using the common-effect inverse-variance model.

Following this, we examined the relationship between initial CEA levels and the effectiveness of ICIs treatment in cancer sufferers. Due to the lack of substantial heterogeneity, a fixed-effects model was utilized (ORR: *I*^2^ = 42.0%, *p* = 0.141; DCR: *I*^2^ = 22.7%, *p* = 0.263; pCR: *I*^2^ = 48.3%, *p* = 0.121). The results showed that individuals with lower CEA levels exhibited higher ORR (OR: 0.53, 95% CI: 0.37–0.78, *p* = 0.001, Figure 4A), DCR (OR: 0.56, 95% CI: 0.43–0.72, *p* < 0.001, Figure 4B) and pCR (OR: 0.58, 95% CI: 0.27–1.23, *p* = 0.060, Figure 4C).

**Figure 5.**
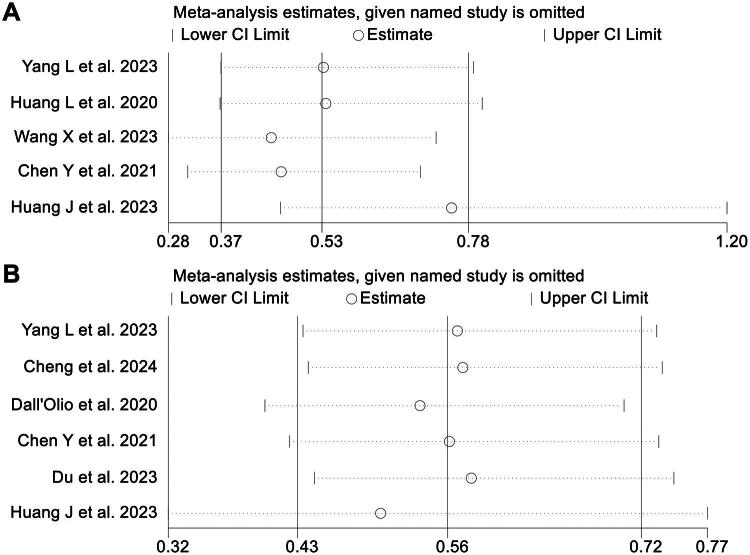
Sensitivity analysis of the relationship between CEA and objective response rate in all included studies (A). Sensitivity analysis of the association between CEA and disease control rate (B). HR: hazard ratio; CL: confidence interval.

The assessment of publication bias was conducted through the application of Begg’s and Egger’s tests. There was an absence of notable bias in publication for ORR (Egger’s test: *p* = 0.730, Begg’s test: *p* = 0.806) and DCR (Egger’s test: *p* = 0.056, Begg’s test: *p* = 0.060). The sensitivity assessments, which entailed repeatedly omitting each study, revealed that the aggregate outcomes for ORR were consistent. The HR calculations for ORR varied between 0.450 (95% CI: 0.280–0.722) after omitting Wang et al. 2023 to 0.747 (95% CI: 0.465–1.202) excluding Huang et al. 2023 (Figure 5A). Likewise, the sensitivity assessments for DCR revealed that the exclusion of any single study had no notable effect on the outcomes, evidenced by HR values varying between 0.500 (95% CI: 0.325–0.770) following the removal of Huang et al. from 2023 to 0.575 (95% CI: 0.446–0.742), following the exclusion of Du et al. 2023 (Figure 5B).

### 3.6. Changed CEA levels and OS and PFS

**Figure 6.**
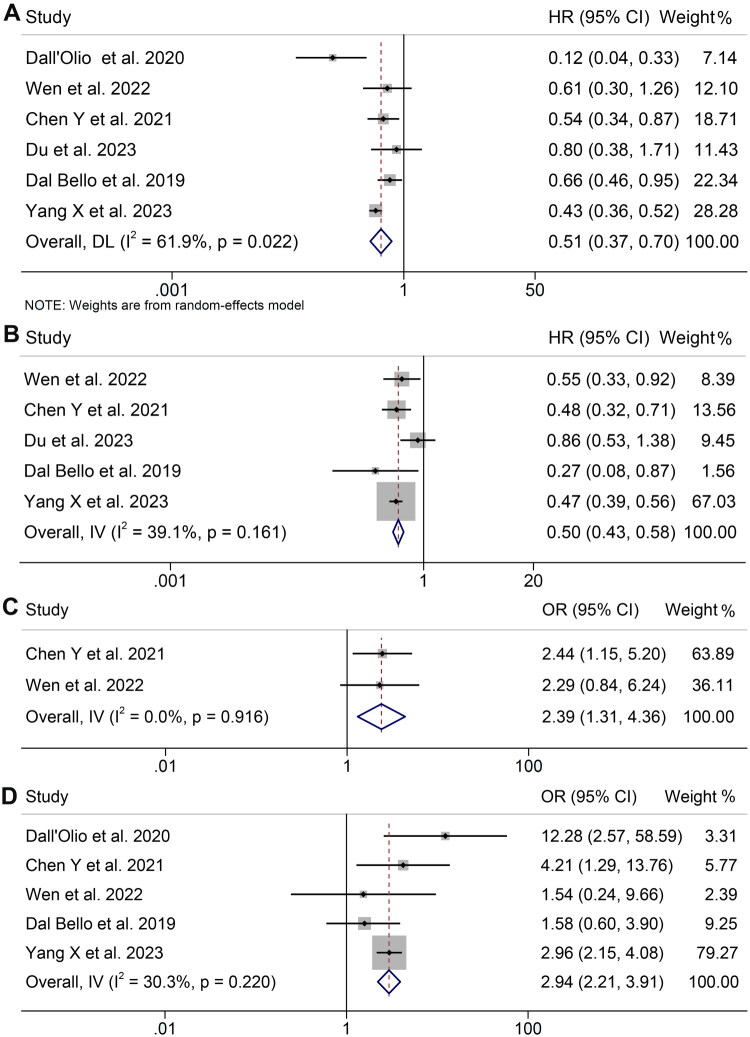
(A) Forest plots of the relationship between changes in CEA levels and overall survival rate in all included studies; meta-analysis pooling of aggregate data using the random-effects inverse-variance model with DerSimonian-Laird estimate of tau [2]. (B) Forest plots of the relationship between changes in CEA levels and progression-free survival in all included studies; meta-analysis pooling of aggregate data using the common-effect inverse-variance model. (C) Forest plots of the relationship between changes in CEA levels and objective response rate in all included studies; meta-analysis pooling of aggregate data using the common-effect inverse-variance model. (D) Forest plots of the relationship between changes in CEA levels and disease control rate in all included studies; meta-analysis pooling of aggregate data using the common-effect inverse-variance model. HR: hazard ratio; CL: confidence interval.

We examined six studies to explore the relationship between alterations in CEA levels and OS during ICIs therapy. Owing to considerable heterogeneity (*I*^2^ = 61.9%, *p* = 0.022), a random-effects model was utilized. The findings revealed a notable correlation between lowered CEA levels and extended overall survival (HR: 0.507, 95% CI: 0.368–0.698, *p* < 0.001, Figure 6A).

Furthermore, the correlation between CEA levels and PFS was analyzed using data from five different studies. Individuals exhibiting decreased CEA levels showed a reduced likelihood of disease progression (HR: 0.501, 95% CI: 0.433–0.581, *p* < 0.001, Figure 6B). Due to the lack of notable diversity (*I*^2^ = 39.1%, *p* = 0.161), a fixed-effects model was employed. The sensitivity assessments, which entailed repeatedly omitting each study, verified the consistency and strength of the combined HRs for both OS and PFS (Figures S2A,B).

### 3.7. Changed CEA levels and ORR and DCR

Additionally, we explored the correlation between changes in CEA levels and ORR and DCR in patients treated with ICIs. Given the absence of notable heterogeneity in the results (ORR: *I*^2^ = 0.0%, *p* = 0.916; DCR: *I*^2^ = 30.3%, *p* = 0.220), a fixed-effects model was used. Our findings revealed that patients with a reduction of CEA levels had a higher ORR (OR: 2.39, 95% CI: 1.31–4.36, *p* = 0.005, Figure 6C) and DCR (OR: 2.94, 95% CI: 2.21–3.91, *p* < 0.001, Figure 6D). Sensitivity analysis confirmed the stability and robustness of the pooled ORRs for DCR (Figure S2C).

## 4. Discussion

The use of ICIs in cancer treatment has gained widespread adoption, prompting extensive research to identify factors influencing their efficacy. The role of CEA in predicting ICI response remains a subject of debate. Our study aimed to address this by synthesizing existing evidence on the relationship between CEA levels and ICI effectiveness. Our analysis reveals that baseline CEA levels are significantly associated with OS, PFS, ORR, DCR, and pCR. Additionally, reductions in CEA levels during immunotherapy were predictive of improved OS, PFS, ORR, and DCR. The robustness of these findings is supported by our publication bias and sensitivity analyses.

The CEACAM-5 gene, belonging to the immunoglobulin superfamily of cell adhesion molecules, encodes CEA, a glycosylphosphatidylinositol (GPI)-anchored glycoprotein on the cell surface [43]. This entity is involved in identifying cells and influences a range of cellular activities. CEA acts as a binding agent for E- and L-selectins, which may affect the likelihood and aggressiveness of metastasis in specific cancer types. This has been linked to the suppression of apoptosis and cellular differentiation in early-stage models [44].

CEA stands as a highly researched and confirmed serum indicator of non-small cell lung cancer (NSCLC) patients, beneficial in diagnosing, predicting outcomes, and evaluating treatments, especially in individuals with mutations in the epidermal growth factor receptor (EGFR) [45]. Yet, the specificity of CEA is not complete, with increased concentrations also appearing in various other cancers, including breast [46], stomach [47], pancreatic [48], cervical [49], and medullary thyroid cancers [50]. Around 35–70% of patients with NSCLC might exhibit elevated CEA levels at the time of diagnosis, particularly in cases involving adenocarcinoma subtypes and EGFR mutations [47]. Increased levels of CEA in the blood have been shown to forecast and predict outcomes in both surgically removed and metastatic NSCLC, with current studies investigating its viability as a biomarker in future studies [51]. Elevated CEA levels in patients with cancer-causing factors are linked to their resistance to EGFR-tyrosine kinase inhibitors (TKIs), underscoring the necessity for more treatment approaches to counteract resistance [52]. For colorectal cancers, a continuous increase in circulating CEA post-surgery correlates with reduced survival rates [53]. Similarly, the presence of metastatic conditions in breast and colon cancer is suggested by serum levels exceeding 20 μg/L [54]. Levels of CEA in the serum are employed to track the advancement of diseases in medullary thyroid and colon carcinomas, and can be utilized if elevated levels are detected in other cancer types [55]. Consequently, despite not being exclusive to a single cancer type, increased expression of CEA is strongly linked to the tumor load in various cancers and indicative of clinical results. It is worth noting that some recent studies have also revealed that CEA can be used as a target for targeted drug delivery, contributing to the treatment of cancer [56,57].

Although the determination of serum CEA concentration in cancer patients is still debated and not yet included in clinical practice guidelines, studies have shown that lower baseline CEA levels are associated with better outcomes in patients receiving immunotherapy. Specifically, Ye et al. found that patients with advanced cholangiocarcinoma who had CEA levels ≤ 5 ng/ml showed better PFS and OS compared to those with CEA > 5 ng/ml [58]. Similarly, Arrieta et al. demonstrated that a reduction in CEA levels during first-line therapy in metastatic NSCLC was associated with prolonged survival and PFS [59]. Consistent with these findings, our study shows that cancer patients undergoing ICI therapy with low serum CEA levels have better OS, PFS, ORR, DCR, and pCR, indicating a more favorable response to immunotherapy. Thus, serum CEA assay emerges as a viable, non-invasive tool for monitoring and prognostic evaluation in cancer patients undergoing immunotherapy.

It is important to note that most of the studies included in this analysis were retrospective cohort studies, which may limit their statistical validity. Furthermore, the variability in the types of ICIs used across studies could affect the consistency of the results. Therefore, further high-quality research is needed, particularly multicenter prospective studies with larger sample sizes, to validate and refine our findings. Additionally, basic research is required to elucidate the mechanisms by which baseline CEA levels influence immunotherapy outcomes and to understand why reductions in CEA levels during treatment are associated with improved outcomes.

## 5. Conclusion

The study underscores the predictive importance of CEA levels concerning the treatment efficacy and clinical results in cancer patients undergoing ICIs. The results advocate for integrating CEA level assessments into the prognostic analysis for these patients.

## Supplementary Material

Supplemental Material

Table S1.docx

## Data Availability

The data that support the findings of this study are available from the corresponding author upon reasonable request (WY).
