# Supplementary material for: Carcinoembryonic antigen as a predictor of treatment outcomes in cancer patients receiving immune checkpoint inhibitors
Source: Ann Med. 2025 Jul 25;57(1):2531255. doi: 10.1080/07853890.2025.2531255 (PMC12302392; doi:10.1080/07853890.2025.2531255)

Figure S1 The picture of the trim-and-fill method terms of overall survival (A) and progression-free survival (B). Theta, the effect estimate; S.e. of: theta, the corresponding standard error.


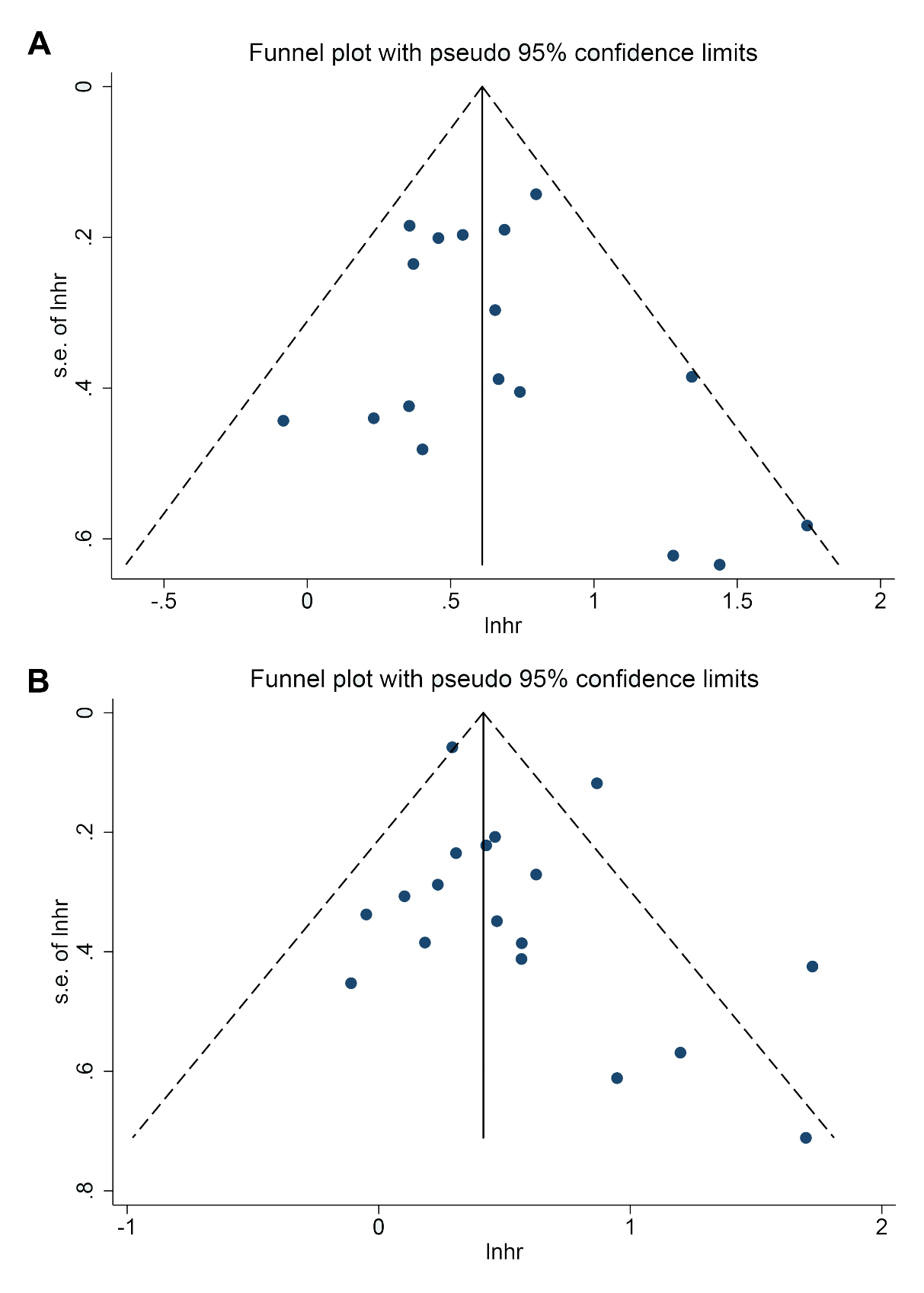


Figure S2 Sensitivity analysis of the association between changes in CEA levels and overall survival rate (A). Sensitivity analysis of the association between changes in CEA levels and progression-free survival (B). Sensitivity analysis of the association between changes in CEA levels and disease control rate (C). HR, hazard ratio; CL, confidence interval.


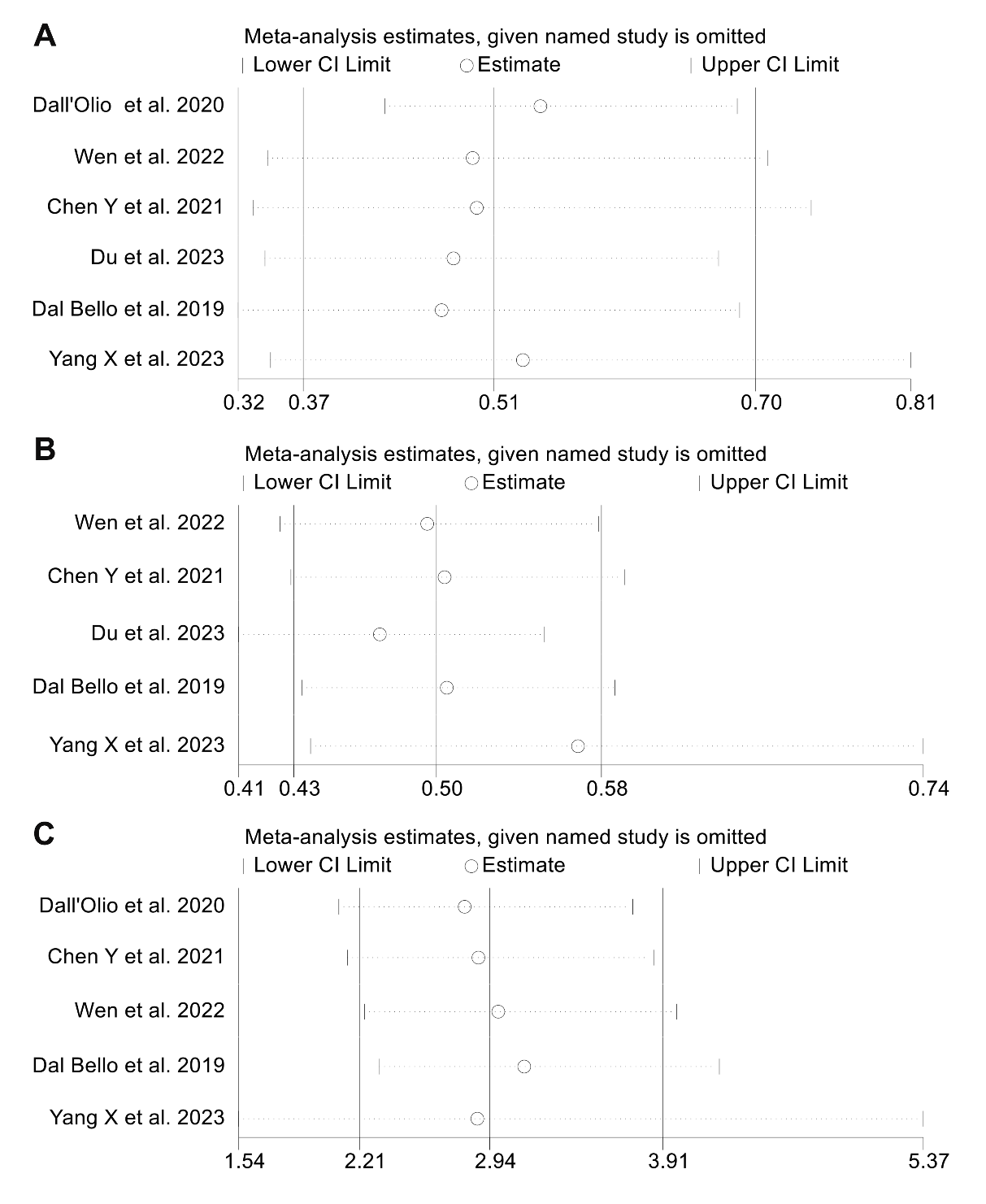

Supplement: Supplemental Material [file IANN_A_2531255_SM4390.docx]
